# Is it time to retire preoperative radiation for localized esophageal and gastro-esophageal adenocarcinoma?

**DOI:** 10.1093/oncolo/oyae371

**Published:** 2025-01-23

**Authors:** Cindy M Pabon, Benjamin Spieler, Jenny J Li, Jaffer Ajani, Peter J Hosein, Mariela Blum Murphy

**Affiliations:** Department of Medicine, Sylvester Comprehensive Cancer Center, University of Miami Miller School of Medicine, Miami, FL 33136, United States; Department of Radiation Oncology, Sylvester Comprehensive Cancer Center, University of Miami Miller School of Medicine, Miami, FL 33136, United States; Department of Gastrointestinal Medical Oncology, The University of Texas MD Anderson Cancer Center, Houston, TX 77030, United States; Department of Gastrointestinal Medical Oncology, The University of Texas MD Anderson Cancer Center, Houston, TX 77030, United States; Department of Medicine, Sylvester Comprehensive Cancer Center, University of Miami Miller School of Medicine, Miami, FL 33136, United States; Department of Gastrointestinal Medical Oncology, The University of Texas MD Anderson Cancer Center, Houston, TX 77030, United States

**Keywords:** esophageal neoplasms, esophageal adenocarcinoma, gastro-esophageal junction adenocarcinoma, preoperative chemoradiation

## Abstract

Whether preoperative chemoradiotherapy (CRT) or perioperative chemotherapy is superior for localized esophageal or gastro-esophageal junction (GEJ) cancers has been a topic of long-standing debate. For years, standard of care in the United States for localized esophageal or GEJ adenocarcinoma (EAC) has been physician’s choice between the 2 strategies. More recently, adjuvant immunotherapy has also been introduced into the treatment approach for those who received neoadjuvant CRT. While preoperative radiation remains an important option for patients with esophageal squamous cell carcinomas, the ESOPEC trial presented in 2024 suggested that perioperative chemotherapy is superior to preoperative CRT in EAC. In addition, the results of the TOPGEAR trial presented in 2024 showed that adding CRT to perioperative chemotherapy did not lead to improved outcomes. This has led to a shift in practice among oncologists. However, there are various complexities and factors to consider when interpreting these studies. In this review, we outline both trials and what their findings may mean for the future of preoperative CRT in EAC. Ultimately, until more data are available that incorporate novel agents such as immunotherapy, these studies indicate that we should defer the routine inclusion of radiation in preoperative treatment for EAC.

**Implications for Practice**

The optimal preoperative strategy for esophageal or gastro-esophageal junction (GEJ) cancers has been a controversial and highly debated topic. Studies presented in ASCO and ESMO conferences, this year suggests that perioperative chemotherapy is superior to preoperative chemoradiation in patients with esophageal or GEJ adenocarcinoma (EAC) histology. Here, we review recent data, noting nuances in trial design, patient population, and study outcomes. We advise that at this point, we should reconsider routine inclusion of radiation in preoperative treatment for EAC. However, in the advent of novel targeted therapies and immunotherapy, we also cautiously await further studies that may revisit the role of preoperative radiation once more.

Esophageal cancer outcomes are poor, with the relative 5-year survival rate for all stages of 21.6%.^1^ For microsatellite stable tumors, esophagectomy is the only curative option, yet around 50% of patients who achieve R0 resection develop recurrence within 2 to 3 years.^2-4^ In efforts to improve outcomes, multimodal strategies have been a focused area of research for locally advanced disease (cT2-4a, cN+/−, cM0). In 2012, the CROSS study established neoadjuvant concurrent chemotherapy and radiation followed by esophagectomy as standard of care for resectable EAC or ESCC.^5^ The neoadjuvant CROSS regimen included weekly carboplatin AUC 2 with paclitaxel 50 mg/m^2^ for 5 weeks and concurrent radiation therapy consisting of 41.4 Gy in 23 fractions. The CROSS regimen resulted in an improved median overall survival (mOS) compared with surgical resection alone (49.4 vs 24 months, HR 0.657; 95% CI, 0.495-0.871, *P* =.003). Long-term follow-up results published in 2015, however, noted a difference in survival based on histology. Specifically, mOS for patients with ESCC was superior at 81.6 months compared with 43.2 months for those with EAC.^6^ Given the worse prognosis in patients with adenocarcinoma, further perioperative approaches continued to be investigated in this group.

In 2006, the MAGIC study led to the implementation of perioperative chemotherapy with epirubicin, cisplatin, and 5-fluorouracil or capecitabine (ECF/ECX) for resectable gastric adenocarcinoma (GAC) or GEJ adenocarcinoma.^7^ In 2019, the FLOT4 study compared 5-fluorouracil, oxaliplatin, and docetaxel (FLOT) to ECF/ECX, demonstrating an improved mOS (50 vs 35 months; HR 0.77; 95% CI, 0.63-0.94).^8^ This led to the adoption of perioperative FLOT as the preferred chemotherapy for resectable GAC or GEJ adenocarcinoma. With this approval, the treatment of choice between FLOT versus CROSS for GEJ adenocarcinoma became unclear. Without head-to-head trials and similar mOS in their respective studies, physician’s practice was largely chosen based on institutional preference and access to radiation services.

The Neoadjuvant Trial in Adenocarcinoma of the Esophagus and Esophago-Gastric Junction International Study (Neo-AEGIS) sought to identify the optimal approach to treat resectable esophageal and GEJ adenocarcinoma. From 2013 to 2020, this phase III open-label randomized study compared the CROSS regimen to perioperative chemotherapy (ECF/ECX or FLOT).^9^ Interestingly, all pathologic outcomes significantly favored CROSS (pCR 16% vs 5%, major pathologic response 32% vs 12%, R0 resection 95% vs 82%, and node negative disease 60% vs 44.5%), but no differences in mOS or PFS were identified between treatment regimens. While Neo-AEGIS was underpowered, its findings suggested that standard of care CROSS versus FLOT remains physician’s choice.

The 2 following trials presented this year, however, may push the needle away from radiation.

## ESOPEC

The ASCO 2024 plenary session released the results from the ESOPEC trial, an investigator-initiated multicenter prospective randomized-controlled study comparing efficacy and survival of preoperative CRT with the CROSS regimen versus perioperative FLOT.^10^ The trial enrolled patients with localized EAC (clinical stages T1N+ or T2-4a,N0/+) but not ESCC, from 2016 to 2020 within 25 centers in Germany. The primary endpoint was OS and secondary endpoints included event free survival, postoperative pathological stage, postoperative complications, recurrence free survival, adverse events, site of tumor recurrence, and quality of life (QOL).

Both the FLOT and CROSS groups were well balanced, with majority of patients being males in their sixties with cT3-4, N+ disease. The FLOT group received 4 cycles of the FLOT regimen preoperatively, and 4 postoperatively, as in the FLOT4 study. The CROSS arm received preoperative carboplatin/paclitaxel concurrent with radiation dosed at 41.4 Gy over 25 fractions, as in the CROSS study. After 4-6 weeks of completing preoperative therapies, patients underwent surgical resection with curative intent.

After a median follow-up of 55 months, it was clear that mOS was superior in the FLOT group (ITT HR 0.70; 95% CI, 0.53-0.92; *P* =.012). Pathological complete response (pCR) as defined by ypT0N0 was also higher in the FLOT group at 16.8% versus 10%. The CROSS pCR was notably lower than what has traditionally been observed in the United States, which may be secondary to differences in radiation dosing. Additional critiques of this study included the lower treatment completion rate in the CROSS group with 67.7% completing neoadjuvant therapy compared with 87.3% in the FLOT group. The lower treatment completion, in addition to the increased 30-day postoperative mortality rates for CROSS (1.7%) versus FLOT (1.0%), may have been influenced by the general familiarity and preference for FLOT in Germany. Nevertheless, the differences in mOS were clear and the median PFS results largely reflected this with a clear separation in the curves favoring FLOT (38 vs 16 months; HR 0.66, 95% CI, 0.51, 0.85; *P* =.001).

For locally advanced EAC patients who receive neoadjuvant CRT but achieve less than pCR, standard treatment now includes a year of adjuvant nivolumab based on outcomes of CHECKMATE-577.^11^ In CHECKMATE-577, disease free survival (DFS) nearly doubled for EAC patients treated with neoadjuvant CRT followed by adjuvant nivolumab (DFS = 19.4 months) versus placebo (DFS = 11.4 months). Therefore, perioperative FLOT versus neoadjuvant CRT+ adjuvant nivolumab represents a more relevant clinical comparison than the ESOPEC randomization. Of note, survival analyses for ESOPEC and CHECKMATE 577 were initiated at different time points: ESOPEC from before frontline treatment, and CHECKMATE 577 from enrollment 4-16 weeks postsurgery. In ESOPEC, the median PFS was 38 months with FLOT versus 16 months with CROSS, whereas for CHECKMATE 577 patients receiving adjuvant nivolumab, DFS was 19 months postenrollment (with 66% of patients [350/532] enrolling at least 10 weeks postsurgery). If starting time points for survival analysis could be synchronized, DFS/PFS outcomes for perioperative FLOT patients in ESOPEC versus CRT patients in the adjuvant nivolumab arm of CHECKMATE-577 appear similar. However, the noninferiority of CROSS+ immunotherapy versus perioperative FLOT has yet to be demonstrated in a prospective trial.

## AGITG TOPGEAR

While the AGITG TOPGEAR trial presented at the ESMO 2024 annual meeting was predominantly a gastric study, its inclusion of GEJ adenocarcinomas allows one to draw further conclusions about the role of preoperative CRT in EAC.^12^ AGITG TOPGEAR was an international, randomized phase III trial in resectable GAC or GEJ adenocarcinoma comparing preoperative CRT plus perioperative chemotherapy (Arm A) or perioperative chemotherapy alone (Arm B). Patients enrolled had cT3-4,N0/+ disease. During the course of the trial, FLOT became approved, leading to an addendum to the protocol to include FLOT as the chemotherapy of choice thereafter. Therefore, Arm A received 2-3 cycles of preoperative ECF/ECX or FLOT, followed by 5 weeks of radiation (45 Gy over 25 fractions) with concurrent continuous 5-fluorouracil infusion or capecitabine. Arm B received 3-4 cycles of preoperative ECF/ECX or FLOT. Both arms underwent surgery 4-6 weeks after preoperative therapy with intended D2 dissection. Both arms also underwent 3-4 cycles of postoperative ECF/ECX or FLOT. Quality assurance for both radiotherapy and surgical techniques was performed.

Both arms were well balanced with predominantly male in their sixties with T3-4 and node positive disease. Given the timeline during which this trial was performed, nearly 70% in both arms received ECF/ECX rather than FLOT. Only about 42%-44% in each arm were able to achieve a D2 dissection at time of surgery and pCR favored Arm A (17% vs 8%, 95% CI, 2-5). However, at the time of median follow-up of 67 months, there was no statistical significant difference in overall survival between the 2 groups with mOS 46-49 months.^13^ This study does have limitations including the inclusion of ECF/ECX and the suboptimal D2 dissection rates. However, one important conclusion that is not influenced by these factors is the finding that tumor downstaging and achieving pCR did not necessarily correlate with an OS advantage. This is similar to recent studies with perioperative immunotherapy in upper GI cancers and serves as a caution to physicians who may be eager to implement changes to practice based on pCR alone. It is important to balance toxicities for the patient and QOL when deciding to pursue complex treatment plans, especially if survival advantage is unclear.

In AGITG TOPGEAR, GEJ adenocarcinoma was defined as “predominantly involving the cardia with no significant esophageal involvement” and included Siewert type III cancers.^13^ Per TNM staging in use at the time of study design and activation, Siewert type III GEJ tumors whose epicenter was up to 5 cm distal to the GEJ were staged as esophageal cancers.^14^ However, the American Joint Committee on Cancer (AJCC) Eight Edition has since redefined the anatomic boundary for staging esophageal versus gastric cancers. According to AJCC v.8, tumors involving the GEJ with epicenter no more than 2 cm into the proximal stomach are now staged as esophageal cancers, whereas tumors whose epicenter is located greater than 2 cm into the proximal stomach are staged as stomach cancers, even if the GEJ is involved.^15^ The current more proximal boundary of the esophagus means that a significant but undefined portion of the GEJ patients enrolled in TOPGEAR (*n* = 98 in the preoperative CRT arm, *n* = 101 in perioperative chemo arm) would no longer be staged as EAC. Therefore clinicians should use caution extrapolating results from GEJ patients in TOPGEAR to guide treatment of current EAC.

## Special populations

Certain subgroups within the EAC population warrant special consideration beyond the general conclusions from the ESOPEC and TOPGEAR trials. This includes patients who may experience difficulty tolerating standard FLOT, such as the elderly. Several studies evaluating perioperative FLOT in patients aged 65 years and above have noted that while FLOT is feasible in this population, it is often at the expense of increased chemotherapy-related toxicity.^16-19^ The FLOT65+ trial analyzed outcomes in elderly patients treated with perioperative FOLFOX versus FLOT, noting a trend toward an improved median PFS with FLOT.^19^ Follow-up QOL studies on this population confirmed an increase in grade 3 and 4 toxicities in the FLOT arm, specifically neutropenia, leukopenia, diarrhea, and nausea. However, QOL measured through the European Organization for Research and Treatment of Cancer Quality of Life Questionnaire C30 (EORTC QLQ-C30) and the gastric module STO22 noted no statistically significant difference in QOL for those treated with doublet versus triplet.^20^ Given that the FLOT65+ trial demonstrated superior efficacy with the triplet, the QOL findings suggest that FLOT should still be pursued in the elderly, though with attention to toxicities and potential need for dose modification. Another consideration could be the possibility of pursuing chemoradiation in this vulnerable group. Jeon and colleagues performed a comprehensive retrospective study (*n* = 57 116) evaluating survival in locally advanced esophageal carcinoma patients treated with varied modalities. They performed a subgroup analyses of patients ≥65 years, noting statistically significant improvement in mOS with perioperative chemotherapy versus other modalities such as perioperative chemoradiation, again supporting the notion that FLOT is feasible and effective in this population.^21^ However, QOL was not addressed in this study. The Neo-AEGIS trial, which showed no statistically significant difference in survival outcomes when comparing trimodality therapy to perioperative chemotherapy, further explored secondary endpoints to guide treatment selection, regardless of age group. These included surgical mortality and morbidity (M&M), toxicity, and Health-Related QOL (HRQOL). The study found no difference in surgical M&M, with a particular focus on common postoperative issues such as pneumonia, respiratory failure, and anastomotic leaks. Interestingly, fewer individuals in the trimodality therapy group withdrew from treatment due to toxicity than those in the perioperative chemotherapy group, though not statistically significant. With respect to HRQOL (following EORTC QLQ-C30), by 1 and 3 years of follow-up, HRQOL differences were largely equal except for an excess of persistent coughing in the trimodality group.^9^ As such, the available data still support perioperative FLOT in vulnerable populations as long as careful monitoring of toxicity is implemented.

An additional group of interest to note is the microsatellite-high (dMMR/MSI-H) population. While this is a unique and relatively small subgroup, a meta-analysis of the MAGIC, CLASSIC, ARTIST, and ITACA-S trials revealed that MSI-H individuals do not benefit from perioperative chemotherapy.^22^ Instead, this population has been found to be exquisitely sensitive to immunotherapy. The NEONIPIGA trial evaluated perioperative immunotherapy with combination nivolumab and ipilimumab, demonstrating a pCR of 58.6%, which is remarkably higher than historical reports for FLOT (7%-20%).^23^ Furthermore, the survival data demonstrated durable response. As such, standard of care for this subgroup has transitioned to an immunotherapy-based approach. The DANTE, MATTERHORN, and KEYNOTE-585 trials have shown that combination of chemotherapy with immunotherapy in the general EAC population can improve pCR results, though survival data as well as subgroup analysis looking at MSI-H patients is still pending.^24-26^ Currently, the literature does not clarify whether adding radiation to immunotherapy in the perioperative setting enhances response in MSI-H EAC. However, the unprecedented pCR rate observed in the NEONIPIGA trial raises the possibility of a nonoperative approach for MSI-H patients, prompting questions about the potential role of consolidative radiation instead of surgery.

Finally, patients with bulky or extensive tumor burden require further multidisciplinary assessment. Thus far, no trial has been powered to assess treatment outcome specific to long or bulky T3 or T4a tumors. In fact, the majority of participants in locally advanced EAC studies tend to have cT3N0-1 disease. Given the low-anticipated pCR rate with standard of care FLOT, it is reasonable to consider other treatment strategies in this bulkier group, including trimodality therapy or novel systemic treatments following FLOT such as adjuvant immunotherapy. The recently published KEYNOTE-585 study did in fact include larger tumors with ≥35% cT4 in both treatment arms. Patients in the intervention arm received neoadjuvant or adjuvant pembrolizumab plus chemotherapy followed by adjuvant pembrolizumab. The study showed a statistically significant 10% improvement in pCR compared with standard of care, although this did not translate to survival benefit and there was no subgroup analysis to outline whether the bulkier tumor group benefited from such strategy.

## Next steps

The debate over the best approach for treating resectable EAC has persisted for a long time. Both Neo-AEGIS and TOPGEAR have now shown that preoperative CRT alone or in addition to perioperative chemotherapy does not improve outcomes in comparison to standard perioperative chemotherapy. With the presentation of ESOPEC, we now favor perioperative FLOT over other strategies. There are several logistic benefits to favor perioperative chemotherapy over chemoradiation. In addition to avoiding common toxicities associated with CRT, such as acute esophagitis, fatigue, and anorexia, perioperative chemotherapy is more accessible to patients who may not be close to a center with radiation capabilities and the treatment schedule is easier to manage.

It is important to note that radiation still has a role in upper gastrointestinal malignancies. The European Society for Medical Oncology (ESMO) guidelines recommend definitive CRT for locally advanced ESCC, with surveillance and salvage esophagectomy when needed for local tumor control even in upfront resectable cases.^27^ Neoadjuvant CRT or definitive CRT remain the National Comprehensive Cancer Network recommendations for treatment of locally advanced ESCC in the United States.^28^ In gastric adenocarcinoma, CRT is standard of care adjuvant treatment for patients who undergo suboptimal curative resection (ie, D1). Macdonald and colleagues demonstrated that this adjuvant approach in patients at high risk of recurrence led to an improvement in mOS (HR 1.35; 95% CI, 1.09-1.66; *P* =.005).^29^ Definitive CRT remains the preferred strategy for patients with gastro-esophageal malignancies who are either unresectable (ie, T4b) or those who are not surgical candidates.^30-32^ Lastly, in the palliative setting, radiation remains the mainstay of treatment for control of subacute tumoral bleeding in gastro-esophageal cancer patients unable to tolerate chemotherapy or who continue to bleed despite receiving systemic therapies. Studies have shown that in addition to controlling the bleed, palliative radiation can provide significant relief of cancer-related pain, dysphagia, and fatigue.^33^ Lastly, chemoradiation may still be beneficial in the special populations noted, such as those with bulky disease, though further investigation is warranted to make such conclusions.

Over the next few years, we anticipate gaining deeper insights into the most effective pre/perioperative strategies as new studies are conducted that explore innovative treatment options such as checkpoint inhibition. It has been suggested that radiation and immunotherapy could work synergistically, potentially reigniting the debate about the role of radiation in the preoperative setting. This theory is backed by the CHECKMATE-577 study, which found that adjuvant nivolumab doubled DFS and decreased the risk of distant recurrence in patients with residual EAC/ESCC at the time of esophagectomy following preoperative CRT.^11^ Potential synergy between CRT and immunotherapy treating intrathoracic malignancy is further supported by impressive OS and PFS results from the PACIFIC trial and others.^34-36^ As such, it is probable that many centers will continue to implement a combination of trimodality therapy with adjuvant immunotherapy.

Ultimately, it is important to obtain timely biomarker testing in EAC and to establish early multidisciplinary discussions on appropriate treatment strategy. Furthermore, as more data are publicized, we must cautiously interpret the pCR and DFS results and await more conclusive survival analyses before changing practice. The varied outcomes of the MATTERHORN, DANTE, and KEYNOTE-585 trials evaluating perioperative immunotherapy for GEJ adenocarcinomas reinforce the idea that survival data will be crucial in prompting changes to treatment strategies for EAC.^24-26,37^ Thus, until more information become available, results from recent studies suggest that we should reconsider routine inclusion of radiation in preoperative treatment for EAC.

## Data Availability

None.
